# Harnessing coaches' expertise: creating 11 sport-specific profiles for talent orientation

**DOI:** 10.3389/fspor.2025.1432903

**Published:** 2025-01-31

**Authors:** Johanna Ochs, Andreas Hohmann, Johan Pion

**Affiliations:** ^1^ Institute of Sport Science, Department of Training and Movement Science, Bayreuth University, Bayreuth, Germany; ^2^ Department of Sport and Exercise Studies, HAN University of Applied Sciences, Nijmegen, Netherlands; ^3^ Faculty of Medicine and Health Sciences, Department of Movement and Sports Sciences, Ghent University, Ghent, Belgium

**Keywords:** coaches, talent, talent detection, sport profiles, talent orientation

## Abstract

**Introduction:**

In Germany, there is no systematic approach to talent orientation that recommends an appropriate sport for children. Talent detection is the first step of the talent process, in which children's motor profiles are assessed using standardized test batteries. In the second step, talent orientation, these profiles are weighted with sport profiles to derive sport recommendations for each child. But how are these sport profiles built? The aim of this study is to engage coaches in the creation of sport profiles.

**Methods:**

German coaches (*n* = 256) of gymnastics, handball, judo, soccer, swimming, table tennis, tennis, track & field participated in a survey using the German Motor Test 6–18 plus ball throwing and agility test. Eight sports were included. Judo was divided into light and heavy weight categories, and track & field into endurance running, sprinting/jumping, and throwing, resulting in eleven disciplines. Each discipline had a separate standardized questionnaire, with judo categories combined into one.

**Results:**

The results show individual profiles of relevant characteristics for each sport discipline. ANOVA and z-transformed means revealed different ratings of the test items, enabling the development of specific combinations of the most important test items for each discipline. The validity of these sport discipline-specific profiles was tested using discriminant analyses, which assigned coaches to their respective sport discipline. A linear discriminant analysis correctly classified 78.1% of coaches to their respective sport discipline. When comparing one sport discipline to all others, correct classification ranged from 82.2% to 92.7%.

**Discussion:**

Based on the coaches' ratings, eleven different sport discipline profiles were developed, each with its own combination of key test items. Track & field sprinting/jumping was most clearly distinguished from other disciplines. Overlaps were found in the profiles of handball and tennis, as well as judo and swimming. These findings help coaches utilize the profiles for talent orientation.

## 1 Introduction

Germany does not have a standardized approach to identifying candidates for achieving the next gold medal. Each sport and each federation search for new talents based on individual concepts. For instance, stand-alone methods are now considered outdated and should be replaced by cooperation between sports federations (1, 2). The different federations initiate their search in clubs, namely with children who already practice this sport. In addition, the children's choice of sport in Germany is not primarily determined by their talent, but by various environmental factors, such as the sport played by their parents. However, a structured process of talent detection (first step) and talent orientation (second step) represents a promising approach to support the match between a child and the practiced sport (2). Therefore, talent detection and talent orientation should be the initial steps in the pathway (3, 4).

Talent detection is the first step (1). Talent detection is the process of testing children for motor skills and physical fitness in order to recognize talents (5). In the majority of cases, talent detection (also referred to as movement checks) is conducted in elementary school, typically between the ages of eight and ten (6–9). Talent detection should take place before puberty, during the period when motor skills are being formed (10, 11). As motor skills remain stable throughout the developmental period, it seems reasonable to provide early guidance to young athletes (10, 12).

The various test batteries utilized for talent detection are exclusively related to motor skills, physical performance, and anthropometric data of children. These batteries are not sport specific and are therefore generic. Examples of internationally recognized test batteries are the Body Coordination Test for Children (13), the EUROFIT Test Battery (14), and the Flemish Sports Compass (15). In Germany, different test batteries are used in various regions. The most widely used test battery is the German Motor Test 6–18 (6, 8, 9, 16–18). Also used are the Emotikon Test Battery (6) and the Fulda Movement Check (19), which have individual extensions or alternative test items. Table 1 provides an overview of the test batteries in Europe and Germany.

**Table 1:** Overview of the test items within the different test batteries in Europe and Germany.

	Body coordination test for children	EuroFit test battery	Flemish sports compass	German motor-test 6–18	Emotikon	Fulda movement check	Survey
20 m sprint				x	x	x	x
30 m sprint			x				
Sideward jumping	x		x	x		x	x
Moving sidewards	x		x				
Balancing backwards	x		x	x		x	x
Flamingo balance		x					
Monopedals skip	x						
Single leg stand					x		
Standing torso bend forward				x		x	x
Push-ups				x		x	x
Knee push-ups			x				
Sit-ups			x	x		x	x
Standing long jump		x	x	x	x	x	x
6-minute endurance run				x	x	x	x
Endurance shuttle run		x	x				
Bicycle ergometer test (PWC 170)		x					
Agility test						x	x
Agility run					x		
10 × 5 m shuttle run test		x	x				
Ball-throwing (80-g ball)						x	x
Medicine ball push					x		
Overhead-throwing test (Badminton shuttle)			x				
Sit-and-reach test		x	x				
Shoulder rotation			x				
Counter-movement jump			x				
Dribbling performance			x				
Hand grip		x				x (new)	
Bent arm hang		x					
Plate tapping		x					

These test batteries serve as a basis for the motor skill and physical performance profiles of children (6, 17). The detection of good movers can be aided by the utilization of test batteries (1), yet it is not possible to make a specific recommendation regarding a particular sport. This is where the second step, talent orientation, comes in. Talent orientation is about giving children a recommendation for a sport in which they can be a talent (5, 15, 20). To do this, it is necessary to combine the child's data from the talent detection with the performance profile of each sport. The sport profiles provide crucial information about the fundamental skills and abilities required for success in the respective sport. They can be used as weighting factors or a given transformation function in the calculation of recommendations (6, 21). The sport profiles can be developed by experts who are very familiar with and proficient in their sport by weighing the skills or test items from the generic test batteries for each sport. Each sport profile can be used to derive a recommendation score using a child's talent detection results. Based on the individual score, each child should be recommended the sports with the highest score.

As coaches, they are talent scouts in practice (22–25) and possess a clear understanding of the requisite physical fitness standards and other relevant criteria (21). Consequently, they can provide insight into the specific characteristics and demands of various sports (26–28). The combination of practical experience and licensure serves to elevate coaches to the status of experts in their field. Thus, coaches have empirical knowledge through their education, experiential knowledge, and necessary professional expertise (29).

Schorer and colleagues (2017) were able to show that regional and national handball coaches exhibited comparable outcomes in talent identification, but unlicensed coaches did not. On the other hand Roberts and colleagues (2020) were able to show that coaches with varying levels of expertise made the same talent judgements (30). Moreover, a survey of racquet sports in which coaches gave different weights to the test items of the Flemish Sports Compass confirmed that coaches are indeed experts (31). Another survey of coaches on the weighting of the test items of the Flemish Sports Compass showed that coaches provided clear weightings, thereby distinguishing paddle sports from other sports (28).

The aim of the present study is to find based on a survey of coaches, the different weightings of the test items used in an annual talent orientation campaign, which lead to specific sport profiles for eleven different sport disciplines and can be used for sport recommendation in the context of talent orientation in Germany. For this purpose, the survey was conducted with 256 coaches from eight sports representing eleven sport disciplines. The hypothesis to be tested is that coaches evaluate the test items in each sport with different degrees of relevance. The eight sports are gymnastics, handball, judo, soccer, swimming, table tennis, tennis, and track & field. In addition, judo was divided into two categories: light weight and heavy weight. Track & field was divided into the three disciplines of endurance running, sprinting/jumping, and throwing.

## 2 Methods

The survey is based on the Fulda Movement Check, a test battery that is closely aligned with the German Motor Test 6–18. All tests in the German Motor Test 6–18 have been subjected to multiple tests for their accuracy (8, 9, 18, 32). The average value for the test-retest correlation is *r_tt_* = 0.82 (16). The Fulda Movement Check extends the test battery by including ball throwing (80-gram ball) and the agility test, which are essential exercises for sport recommendations (17). The test-retest correlation for the ball throwing test is *r_tt_* = 0.82 (17). The values for the agility test, as determined by the authors' own data are *r_tt_* = 0.784 (*n* = 129, *p* < .001) for boys and *r_tt_* = 0.891 (*n* = 134, *p* < .001) for girls.

A total of 256 coaches (209 male, 41 female, 3 divers and 3 with no information) from all over Germany were included in an online Qualtrics survey to reflect the perspectives of various regions (33). The respondents had a mean age of 45.32 (±11.49) years and a mean experience of 18.16 (±11.52) years. Forty coaches hold an international license or the highest possible license in their sport. One hundred and sixty coaches hold a national license, 31 coaches hold a regional license, and 10 coaches hold a club license. Fifteen coaches could not be assigned to a license level or did not have a license. The number of coaches in the following sport disciplines was as follows: 18 in gymnastics, 28 in handball, 23 in judo light weight and heavy weight categories, 31 in soccer, 47 in swimming, 21 in table tennis, 25 in tennis, 19 in track & field endurance running, 29 in track & field sprinting/jumping and 15 in track & field throwing. The coaches were engaged in practice of coaching at the time of the survey and were recruited through the national professional associations and personal approaches.

The questionnaire was designed with a standardized structure and was created in ten sport-specific versions. Sport specific means that in each question, the participants were reminded to answer based on their respective sport or discipline. All other wording was identical. Judo coaches completed two relevance rankings, one for light weight category judo and one for heavy weight category judo, in one version. The first page contained a brief description of the research project, followed by descriptions of the test items, including pictures and written explanations. Following the description of the ten tests (one per page), the coaches were asked to rate the relevance of the tests to their sport on a scale of 0–100, with 0 indicating that the test was not relevant and 100 indicating that it was highly relevant. Furthermore, the data were transformed to a scale of 10 by dividing all values by 10 and rounding to the nearest integer. Finally, the coaches were asked to provide information about their sociodemographic characteristics (e.g., age, gender) as well as key characteristics of their coaching role (e.g., license, years of experience).

A total of 279 coach responses were initially analyzed. However, 46 responses were excluded from further analysis because more than two test items were not rated by the coaches. In 39 of the remaining 233 responses a maximum of two test items were not assessed. In these cases, the mean rating of the test item across all participants from the same sport was used instead. Therefore, 233 responses were included in the following analysis.

SPSS version 28.0.1.0 (142) was used for data analysis. The data has been anonymized. Reliability for internal consistency was tested using Cronbach's alpha, and the results can be confirmed as given (acceptable to very good) (34): gymnastics = 0.677, handball = 0.807, judo light weight category = 0.783, judo heavy weight category = 0.781, soccer = 0.916, swimming = 0.861, table tennis = 0.795, tennis = 0.742, track & field endurance running = 0.842, track & field sprinting/jumping = 0.838, track & field throwing = 0.743. The Intraclass Correlation Coefficient was used to calculate the interrater reliability and can be considered at least moderate to good in all sports (35): gymnastics = 0.489, handball = 0.738, judo light weight category = 0.722, judo heavy weight category = 0.662, soccer = 0.778, swimming = 0.812, table tennis = 0.692, tennis = 0.536, track & field endurance running = 0.756, track & field sprinting/jumping = 0.613, track & field throwing = 0.627.

First, descriptive statistics (means and standard deviations) were performed. Then, a Welch-ANOVA with a Games-Howell *post hoc* test was performed to test for similarities and differences between the sports for each test item, with the aim of identifying significant differences. To mitigate potential chance capitalization, the *p*-values from the Welch-ANOVA results were transferred to R and recalculated using the False Discovery Rate method by Benjamini and Hochberg (36). The values of a test item and a sports discipline were compared.

In order to make the differences and similarities of the individual sports disciplines comparable, the data were z-standardized. For this purpose, the mean and the standard deviation were calculated of all data.

To further clarify the differences between the sport disciplines, two discriminant analyses were carried out. In the first discriminant analysis (DA), each sport discipline was discriminated against all other sport disciplines separately. In the second one, all sport disciplines were discriminated against each other. To ensure an even distribution of data across the various sport disciplines, subgroups of a similar size were systematically formed based on the *a priori* classification probability. These subgroups were used to calculate the DA, and the process was repeated until all cases were included. All further calculations were based on the mean value of the DAs per sport discipline.

## 3 Results

### 3.1 Sport profiles: descriptive statistics

First, descriptive statistics show the means and standard deviations (Table 2). The test items were rated differently in the various sport disciplines. Across all sport disciplines and test items the minimum value is 1.43 ± 1.8 (80-gram ball throwing—soccer) and the maximum value is 8.73 ± 2.07 (20 m sprint—track & field sprinting/jumping).

**Table 2:** Descriptive statistics with mean scores, standard deviation and ANOVA results for all test items and sport disciplines.

	20 m Sprint	Balancing backwards	Sideward jumping	Sit-ups	Push-ups	Standing long jump	Standing torso bend forward	6-minute endurance run	Ball-throwing (80-gram ball)	Agility test
Gymnastics	8.07 ± 1.62**^d,^ *^f,^ *^g^	6.13 ± 2.42*^i^	5.80 ± 1.97	6.47 ± 2.36**^e,^ *^j^	6.33 ± 2.53**^e,^ *^j^	6.73 ± 1.58*^e^	8.20 ± 1.52**^b,^ **^e,^ **^g,^ **^h,^ **^i,^ **^j, *k^	4.20 ± 2.18*^i^	2.80 ± 2.25**^b,^ **^h,^ **^k^	5.27 ± 2.40*^c,^ *^h^
Handball	7.00 ± 1.98**^d^	4.44 ± 2.95	6.52 ± 1.85*^d,^ *^e^	4.84 ± 2.72**^e^	5.60 ± 2.60**^e,^ *^j^	6.24 ± 2.35*^e^	4.42 ± 2.47**^a,^ *^e,^ *^f^	5.24 ± 2.18*^i,^ *^j^	8.20 ± 1.26**^a,^ **^c,^ **^d,^ **^e,^ **^f,^ **^g,^ **^i,^ **^j^	7.08 ± 2.06*^f^
Judo light weight category	5.32 ± 3.22*^j^	5.79 ± 3.05	5.63 ± 2.34	6.47 ± 2.32**^e,^ *^h,^ *^i,^ **^j^	6.95 ± 2.32**^e,^ *^g,^ *^i,^ **^j^	6.53 ± 2.55*^e^	6.53 ± 2.99**^e,^ *^i,^ *^j^	6.58 ± 2.57**^j^	3.38 ± 3.05**^b,^ **^h,^ *^k^	8.32 ± 1.34*^a,^ **^f,^ *^j,^ **^k^
Judo heavy weight category	3.30 ± 2.39**^a,^ **^b,^ **^e,^ **^h,^ *^i,^ **^j,^ *^k^	4.90 ± 2.49	4.30 ± 1.72*^b,^ **^g,^ *^h^	5.80 ± 2.19**^e,^ **^j^	6.75 ± 2.05**^e,^ *^g,^ *^i,^ **^j^	5.05 ± 2.35*^h^	5.55 ± 3.22*^e^	4.90 ± 2.20*^i,^ *^j^	3.25 ± 3.21**^b,^ **^h,^ *^k^	6.80 ± 2.02
Soccer	7.88 ± 1.88**^d,^ **^f,^ **^g^	3.88 ± 3.10	3.64 ± 3.03*^b,^ **^g,^ *^h,^ *^j^	1.65 ± 1.79**^a,^ **^b,^ **^c,^ **^d,^ **^f,^ *^g,^ *^h,^ **^k^	1.62 ± 1.72**^a,^ **^b,^ **^c,^ **^d,^ **^f,^ **^h,^ *^i,^ *^k^	3.73 ± 2.65*^a,^ *^b,^ *^c,^ **^f,^ **^h,^ *^j,^ **^k^	2.27 ± 1.71**^a,^ *^b,^ **^c,^ *^d,^ **^f,^ **^h,^ **^k^	4.77 ± 3.17*^i^	1.43 ± 1.80**^b,^ *^g,^ **^h,^ **^k^	6.29 ± 2.61
Swimming	5.21 ± 2.75*^a,^ *^e,^ **^j^	4.28 ± 2.84	4.76 ± 2.65*^g^	6.67 ± 2.38**^e,^ *^g,^ *^h,^ *^i,^ **^j^	7.09 ± 2.75**^e,^ **^g,^ *^i,^ **^j^	7.12 ± 2.61**^e^	6.94 ± 2.69*^b,^ **^e,^ *^g,^ **^i,^ **^j^	5.85 ± 2.60**^j^	2.92 ± 2.99**^b,^ **^h,^ **^k^	4.81 ± 2.75*^b,^ **^c,^ *^g,^ **^h^
Table tennis	4.61 ± 2.55*^a,^ *^e,^ **^j^	4.61 ± 2.59	7.33 ± 1.78**^d,^ **^e,^ *^f^	4.17 ± 2.46*^e,^ *^f^	3.53 ± 1.97*^c,^ *^d,^ **^f,^ *^h^	5.71 ± 2.62	3.83 ± 2.01**^a,^ *^f^	4.06 ± 2.39**^i^	4.78 ± 2.37**^b,^ *^e,^ **^h,^ *^j,^ *^k^	7.50 ± 1.79*^f^
Tennis	7.00 ± 1.91**^d^	4.74 ± 2.47	6.57 ± 2.02*^d,^ *^e^	4.00 ± 2.38*^c,^ *^e,^ *^f^	5.83 ± 1.86**^e,^ *^g,^ **^j^	7.29 ± 1.27*^d,^ **^e,^ *^i^	5.08 ± 1.93**^a,^ **^e^	4.92 ± 2.60*^i,^ *^j^	8.25 ± 1.89 **^a,^ **^c,^ **^d,^ **^e,^ **^f,^ **^g,^ **^i,^ **^j^	8.13 ± 0.95*^a,^ **^f,^ *^j,^ **^k^
Track & field endurance running	6.61 ± 2.81*^d^	2.50 ± 2.77*^a^	5.00 ± 2.47	3.72 ± 2.37*^c,^ *^f^	3.89 ± 2.11*^c,^ *^d,^ *^e,^ *^f^	4.78 ± 2.49*^h,^ *^k^	3.11 ± 2.32**^a,^ *^c,^ **^f^	7.94 ± 2.24*^a,^ *^b,^ *^d,^ *^e,^ **^g,^ *^h,^ **^j,^ *^k^	3.27 ± 2.81**^b,^ **^h,^ *^k^	6.17 ± 2.38
Track & field sprinting/jumping	8.73 ± 2.07*^c,^ **^d,^ **^f,^ **^g^	3.55 ± 2.34	6.23 ± 2.14*^e^	2.95 ± 2.17*^a,^ **^c,^ *^d,^ **^f^	2.55 ± 1.99*^a,^ *^b,^ **^c,^ **^d,^ **^f,^ **^h,^ *^k^	6.77 ± 2.69*^e^	3.45 ± 2.15**^a,^ *^c,^ **^f^	2.36 ± 2.38*^b,^ **^c,^ *^d,^ **^f,^ *^h,^ **^i^	1.71 ± 1.76**^b,^ *^g,^ **^h,^ **^k^	5.45 ± 3.02*^c,^ *^h^
Track & field throwing	7.38 ± 2.36*^d^	5.38 ± 2.33	4.54 ± 2.40	4.92 ± 1.85**^e^	5.69 ± 2.43*^e,^ *^j^	7.54 ± 1.85**^e,^*^i^	5.50 ± 1.68*^a,^ **^e^	4.23 ± 2.17*^i^	8.23 ± 2.49**^a,^ *^c,^ *^d,^ **^e,^ **^f,^ *^g,^ *^i,^ **^j^	5.62 ± 1.33**^c,^ **^h^

*^a^ Significant difference with gymnastics (*p* < 0.05).

**^a^ Significant difference with gymnastics (*p* < 0.001).

*^b^ Significant difference with handball (*p* < 0.05).

**^b^ Significant difference with handball (*p* < 0.001).

*^c^ Significant difference with judo light weight category (*p* < 0.05).

**^c^ Significant difference with judo light weight category (*p* < 0.001).

*^d^ Significant difference with judo heavy weight category (*p* < 0.05).

**^d^ Significant difference with judo heavy weight category (*p* < 0.001).

*^e^ Significant difference with soccer (*p* < 0.05).

**^e^ Significant difference with soccer (*p* < 0.001).

*^f^ Significant difference with swimming (*p* < 0.05).

**^f^ Significant difference with swimming (*p* < 0.001).

*^g^ Significant difference with table tennis (*p* < 0.05).

**^g^ Significant difference with table tennis (*p* < 0.001).

*^h^ Significant difference with tennis (*p* < 0.05).

**^h^ Significant difference with tennis (*p* < 0.001).

*^i^ Significant difference with track & field—endurance running (*p* < 0.05).

**^i^ Significant difference with track & field—endurance running (*p* < 0.001).

*^j^ Significant difference with track & field—sprinting/jumping (*p* < 0.05).

**^j^ Significant difference with track & field—sprinting/jumping (*p* < 0.001).

*^k^ Significant difference with track & field—throwing (*p* < 0.05).

**^k^ Significant difference with track & field—throwing (*p* < 0.001).

The red-marked symbols will no longer appear significant after applying the False Discovery Rate.

### 3.2 Differences: ANOVA

Second, the assessments of coaches regarding test items vary between the different sport disciplines. Table 2 illustrates this. Therefore, for each sport discipline, there is a specific combination of test items in a specific order, which depends on the relevance of the individual test items. It is noteworthy that the track & field disciplines, namely endurance running and sprinting/jumping, differ only in the test item 6-min endurance run, which is significantly different (*p* < .001) from each other. Handball does not differ significantly from tennis and track and field throwing in any of the test items. Tennis and track & field throwing are significantly different (*p* < .001) from each other in the agility test. This fact is similar for judo heavy weight category to judo light weight category and swimming.

In addition, there are test items in the respective sport discipline that differ from many other sport disciplines. For instance, the test item standing long jump in soccer differs from seven other sport disciplines. This phenomenon of significant differences accumulating in all sport disciplines is evident in all cases. A specific combination of high-ranked test items exists for each sport discipline, which is not identical to any other sport disciplines. It becomes evident that these combinations of test items constitute the most valid recommendation tool.

**Figure 1:**
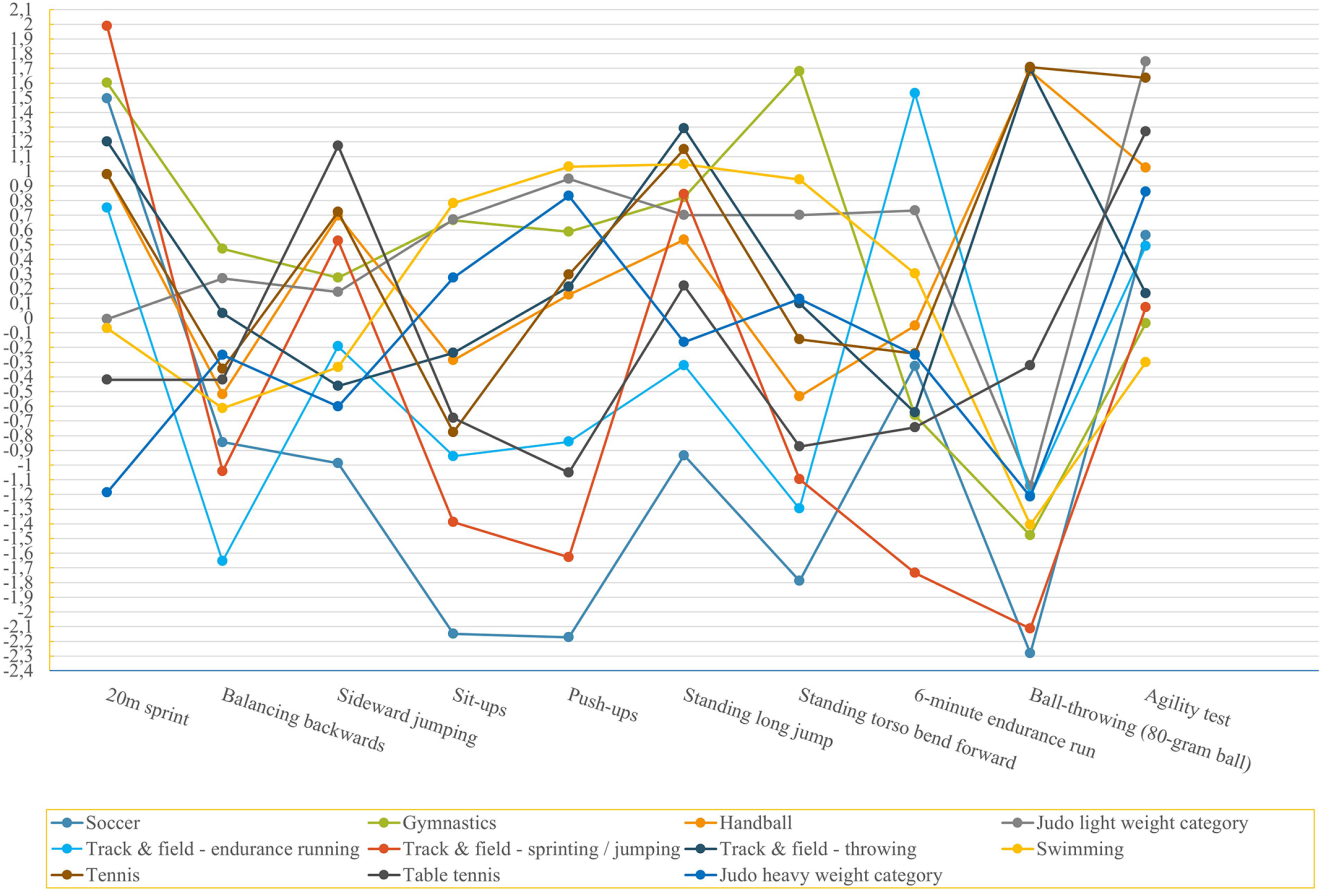
The z-transformed means of coaches’ assessment show how differently relevant the test items were rated between the sport disciplines.

The z-transformed means from the coaches’ assessments in Figure 1 illustrate this. For the three most important test items of each sport discipline it can be stated that for at least one test item a difference occurs between the discipline and another sport discipline, as can be seen from the ANOVA. Furthermore, for each sport discipline at least one test item is significantly different from four or more other sport disciplines.

### 3.3 Classification: discriminant analysis

a) One sport discipline against all other sport disciplines

The percentage of correctly assigned coaches for each sport is as follows: soccer (92.7%), track & field sprinting/jumping (92.5%), gymnastics (91.2%), tennis (89.8%), table tennis (89.3%), judo heavy weight (88.9%), track & field throwing (88.9%), track & field endurance running (87.9%), swimming (87.7%), handball (86.8%), and judo light weight (82.2%).

b) All sport disciplines against all other sport disciplines

The results of the first discriminant analysis (DA) were supported by the use of only two grouping variables. By contrasting all sport disciplines with all other sport disciplines, the linear DA shows that 78.1% (182 of 233 cases) of the sport specific profiles, weighted by the coaches, could be correctly assigned to their respective sport disciplines.

In the track & field sprinting/jumping, 95.5% (*n* = 21) were correctly classified. The other case was assigned to table tennis (*n* = 1).

92.3% (*n* = 12) of the track & field throwing cases were correctly classified, the other case was classified as handball (*n* = 1).

The cases related to swimming are correctly classified by 87.9% (*n* = 29). Furthermore, one case each is classified in handball (*n* = 1), judo light weight category (*n* = 1), judo heavy weight category (*n* = 1) and track & field sprinting/jumping (*n* = 1).

The tennis cases are correctly classified as 83.3% (*n* = 20). The remaining cases are classified in handball (*n* = 3) as well as track & field sprinting/jumping (*n* = 1).

In handball, 80.0% (*n* = 20) are correctly classified. The other cases are classified in tennis (*n* = 4) and table tennis (*n* = 1).

76.9% (*n* = 20) of the soccer cases are correctly classified by the linear DA, the other cases are classified in handball (*n* = 1), judo light weight category (*n* = 1) as well as swimming (*n* = 1), table tennis (*n* = 1), tennis (*n* = 1) and track & field sprinting/jumping (*n* = 1).

In table tennis 72.2% (*n* = 13) are correctly classified. The other cases are classified as handball (*n* = 1), judo heavy weight category (*n* = 2), swimming (*n* = 1) and track & field throwing (*n* = 1).

For the track & field endurance running, 72.2% (*n* = 13) cases were correctly classified. The remaining cases were classified as soccer (*n* = 2), swimming (*n* = 2) and tennis (*n* = 1).

In gymnastics, 66.7% (*n* = 10) are classified to their sport discipline, the other cases are classified to track & field sprinting/jumping (*n* = 1) as well as swimming (*n* = 4).

In the judo light weight category, 63.2% (*n* = 12) of the cases are correctly classified. The other cases are spread in, handball (*n* = 1), judo heavy weight category (*n* = 3), swimming (*n* = 1) and table tennis (*n* = 2).

Judo heavy weight category cases are correctly classified by 60.0% (*n* = 12). 15% of the cases are classified as judo light weight category (*n* = 3) and 15% of the cases are classified as swimming (*n* = 3), the remaining cases are classified as handball (*n* = 1) and track & field endurance running (*n* = 1).

Despite the use of eleven grouping variables a correct classification of 78.1% is achieved in the linear DA. When the same procedure is applied to only one of the judo disciplines, the result improves to 79.8%.

**Figure 2:**
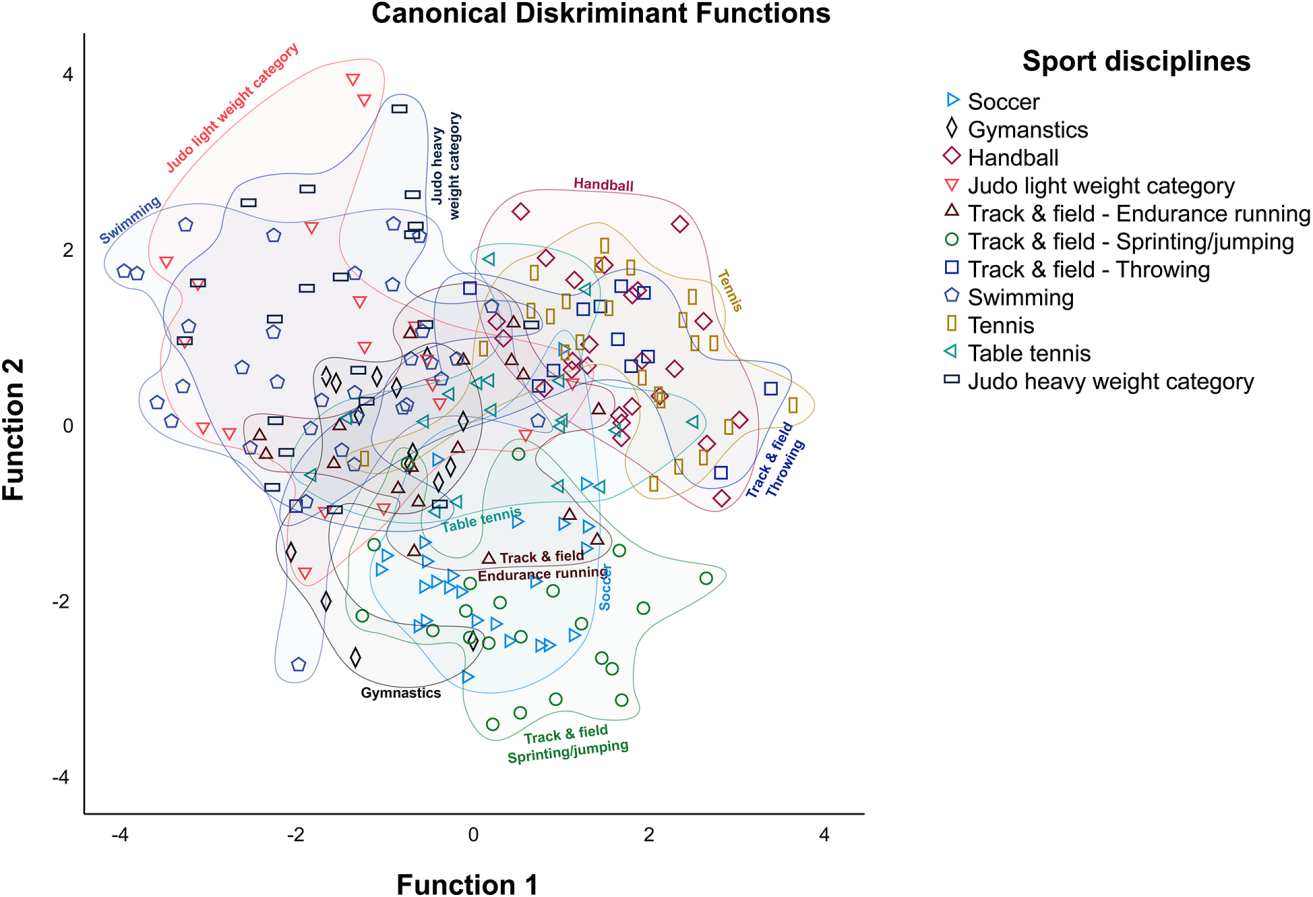
Graphic result of canonical discriminant functions of the eleven different sport disciplines.

Figure 2 corroborates the results presented in the preceding section. The chart illustrates the different and similar responses of coaches in their respective sport disciplines, as indicated by the colored areas. The groups of gymnastics, soccer, table tennis, and track & field endurance running as well as track & field sprinting/jumping have their own center as the sport specific group of coaches provided a homogeneous weighting of the relevant test items. In contrast, the coaches from handball, tennis, and track & field throwing are located close together. The chart shows a large overlap between the groups. Furthermore, the judo light and heavy weight categories and swimming are in proximity to each other. Figure 2 shows that there are outliers in the response behavior of coaches in the sport disciplines swimming, gymnastics, judo light weight and heavy weight categories and track & field endurance running that differ from the rest of the group. In the case of these outliers, it is likely that they were incorrectly assigned to another sport in the DA.

## 4 Discussion

This study, based on a coaches' survey on the weighting of the ten test items, showed that the coaches' weighting resulted in profiles of eleven sport disciplines. The results of the ANOVA (Table 2), z-transformation (Figure 1), and the DA revealed differences and similarities among the eleven sport disciplines from the coaches' perspective. It became evident that each sport discipline has a unique combination of test items that represent the essential skills of the sport discipline. This also confirms the distinction between the individual sports (disciplines) profiles on the non-sport specific test battery as shown in the survey (21, 28). So, it is feasible to find the relevant performance characteristics for each sport (discipline) from a generic test battery (37).

The results of the DA indicate that 78.1% of the coaches' profiles were correctly allocated to their respective sport disciplines. This shows that all eleven sport disciplines can be distinguished from each other.

The differentiation between track & field sprinting/jumping and track & field throwing was most apparent, with 95.5% and 92.3% of coaches correctly classifying these disciplines, respectively. Track & field sprinting/jumping with the 20 m sprint being rated particularly highly as the most important test item and for track & field throwing, it's the ball throwing test item (Table 2).

In certain sport disciplines, individual test items are more prominent than in others. However, the assignments of the coaches are in these sport disciplines as less clear than in the aforementioned sport disciplines. This is evident in gymnastics, soccer, track & field endurance running and table tennis. In gymnastics, the torso bend forward test item is a unique differentiator compared to other sport disciplines. In soccer, only 20 m sprint and the agility test item were identified as relevant test items, with all other test items being rated very low compared to the other sport disciplines. The 6-minute endurance run is a distinguishing feature of track & field endurance running. In the sport of table tennis, the test item sidewards jumping is particularly noteworthy.

Swimming is distinguished from other sport disciplines by a high score in test items on the abilities of strength, stability, and flexibility of the core. Judo's two weight categories are the closest to swimming, also scoring high on the associated test items. In this study, handball, tennis, and track & field throwing are distinguished from all other sport disciplines by the high relevance of the ball throwing test items. They can be differentiated from each other by the different evaluation of the other test items (Figure 1).

Based on an individual number of significant differences between the sport disciplines regarding the test items, each sport discipline can be described by a specific profile (21). In conclusion, each validated profile in this study can be described as a specific profile and can therefore be used in the context of talent orientation.

Different variables are required in different sport disciplines to constitute specific profiles. In addition to these findings, the DA (Figure 2) indicates that sport disciplines have similarities and differences. The similarities are due to an overlap in the key talent characteristics (21). Therefore, one finding in this sample is that handball and tennis are similar. A total of 12.5% (*n* = 3) of the tennis coaches were classified as handball coaches, while 16.0% (*n* = 4) of the handball coaches were classified as tennis coaches. The other sport was assigned to these coaches as the “true” sport due to the overlap of ball throwing as an important characteristic in the sport profile. These findings align with those of Teunissen et al. (2021), who show that the characteristics of handball and tennis were perceived as similar. In this study, another sport discipline with some overlaps with tennis and handball is track & field throwing. Ball throwing is also a crucial test item for this sport discipline. The other items help to differentiate track & field throwing.

Another result of the study is a large overlap between judo (in general) and swimming. This overlap is particularly evident in the push-up test item. This test item is rated as particularly important and similar in both sports. Additionally, tennis and table tennis exhibit some overlap. These findings are consistent with Robertson et al. (2018), who show that the sport profiles of racquet sports are similar. These results are possible because there is a general similarity between the sports (38).

Furthermore, if the DA is calculated with only one judo category, the result increases to 79.8%. This demonstrates the similarity in the response behavior of the judo coaches. It can be concluded that the requirements for motor skills and physical fitness in judo at a young age are similar in the different weight categories. Additionally, the samples of judo coaches are not independent samples. It is possible that the experts gave almost the same answers. In retrospect, it is not necessary to differentiate between the weight classes in judo.

The results of the study indicate the potential for a specialized sampling strategy (33) or a task-related sampling (39) in the early phase of talent orientation as well as for later talent transfer (2). Overall, not all athletes are talents in their originally chosen sport; however, their profile may be better suited to other sports. These findings may assist athletes in transitioning to a sport that aligns with their abilities (21).

It is clear that coaches are well positioned to assess the aforementioned weightings and should be included in the development of talent identification scores (21, 27). This can also be applied to scores for sport recommendations. The mapping in the discriminant analysis and the standard deviations of the descriptive results show that coaches do not always agree with each other. This finding is confirmed by Roberts in a study on talent identification (30).

The development of sport discipline profiles provides coaches with a tool to assess whether a child is talented in a particular sport. They can utilize this information to recommend the most promising candidates for a/their sport. To achieve this, the results of children who have completed the test items can be compared with the sport disciplines profiles. This comparison can be based on a weighting procedure that calculates a composite score for each child, reflecting their suitability for various sports. These scores can then be used to rank the children, enabling a clear and systematic identification of the most appropriate sports for each individual.

Additionally, children receive an indication of what skills they need to improve to become more successful. Occasionally this method can indicate whether other sports may be more promising for the child than the actual sport. This is why talent orientation is an important tool for providing children with the opportunity to try new sports (1). Regarding the screening of a larger number of children to detect potential talents, the results strengthen the importance of talent orientation, including movement checks and cross-sport checks.

This study is not without limitations. One such limitation is that it was not asked whether the coaches in question train boys or girls. It might be possible that this influences the response behavior, for example in gymnastics, and should be considered in future research. It should also be noted that the two rankings in the light and heavy weight categories of judo do not originate from independent samples, in contrast to all other sport disciplines. The same coaches train different weight categories in the youth. With the inclusion of further sports, the separation of profiles becomes increasingly challenging, despite the inclusion of eleven sport disciplines in this study (28). The sports considered here are those included in the sports recommendation of the regional Fulda Movement Check campaign in the Fulda region (Germany), which offers children the opportunity to exercise their talents locally (19).

In addition, further research is needed to ascertain whether the sport recommendations derived from this study (e.g., the Fulda Movement Check) are consistent with the individual decisions made (26). The question arises as to which factors are relevant in this regard. Furthermore, the number of sports (disciplines) should also be increased in the context of this survey. In addition, the results can be strengthened or refuted through expert interviews. A comparison with the current guidelines of the sports federations can also reinforce the results. Moreover, it is important to consider these rankings in the context of the applicable rules, since changes in the rules of a given sport may alter the physical demands from the outset (i.e., at the point of entry into the sport) (40).

In conclusion, 11 sport specific profiles were developed in this study using a coach survey. Due to their discriminative validity, these profiles can be utilized, in conjunction with other factors, as a tool for individual sport recommendation in talent orientation programs. Concurrently, they can be used to support talent transfer measures based on the similarity of sports. The study thus contributes to the field of talent orientation in youth sports based on an expert survey of coaches and a non-sport specific test battery.

## Data Availability

The datasets presented in this article are not readily available because the dataset is part of ongoing research. Requests to access the datasets should be directed to Johanna Ochs, joh.ochs@gmail.com.

## References

[1] Pion J. From Potential to Performance: The Power of Tools for Talent Identification and Development. Ghent: Hylyght (2024).

[2] Pion J Teunissen JW Ter Welle S Spruijtenburg G Faber I Lenoir M. How similarities and differences between sports lead to talent transfer. In: Baker J Cobley S Schorer J, editors. Talent Identification and Development in Sport. London: Routledge (2020). p. 184–96.

[3] Hohmann A Seidel I. Scientific aspects of talent development. Int J Phys Educ. (2003) 40:9–20.

[4] Williams AM Reilly T. Talent identification and development in soccer. J Sports Sci. (2000) 18:657–67. 10.1080/02640410050120041 11043892

[5] Vaeyens R Lenoir M Williams AM Philippaerts RM. Talent identification and development programmes in sport current models and future directions. Sports Med. (2008) 38:703–14. 10.2165/00007256-200838090-00001 18712939

[6] Ester J Zinner J Utesch T Büsch D. Nutzung multikriterieller und unscharfer (FUZZY-)analysen zum talentscreening im sport. Informatik Spektrum. (2020) 43:103–17. 10.1007/s00287-020-01251-w

[7] Fuchslocher J Romann M Rüdisüli R Birrer D Hollenstein C. Das talentselektionsinstrument PISTE: wie die Schweiz Nachwuchsathleten auswählt. Leistungssport. (2011) 41:22–7.

[8] Golle K Muehlbauer T Wick D Granacher U. Physical fitness percentiles of German children aged 9–12 years: findings from a longitudinal study. PLoS One. (2015) 10:e0142393. 10.1371/journal.pone.0142393 26544848 PMC4636306

[9] Hohmann A Siener M. Talent identification in youth soccer: prognosis of U17 soccer performance on the basis of general athleticism and talent promotion interventions in second-grade children. Front Sports Act Living. (2021) 3:625–45. 10.3389/fspor.2021.625645 PMC8212928 34151260

[10] Fransen J Pion J Vandendriessche J Vandorpe B Vaeyens R Lenoir M Differences in physical fitness and gross motor coordination in boys aged 6–12 years specializing in one versus sampling more than one sport. J Sports Sci. (2012) 30:379–86. 10.1080/02640414.2011.642808 22214429

[11] Watanabe D Savion-Lemieux T Penhune VB. The effect of early musical training on adult motor performance: evidence for a sensitive period in motor learning. Exp Brain Res. (2007) 176:332–40. 10.1007/s00221-006-0619-z 16896980

[12] Vandorpe B Vandendriessche JB Vaeyens R Pion J Lefevre J Philippaerts RM The value of a non-sport-specific motor test battery in predicting performance in young female gymnasts. J Sports Sci. (2012) 30:497–505. 10.1080/02640414.2012.654399 22263781

[13] Kiphard EJ Schilling F. Körperkoordinationstest für Kinder KTK. Weinheim: Beltz Test GmbH (1974).

[14] Council of Europe. Eurofit: Handbook for the EUROFIT Tests of Physical Fitness. Rome: Secretariat of the Committee for the Development of Sport within the Concil of Europe (1988).

[15] Pion J. The Flemish Sports Compass: From Sports Orientation to Elite Performance Prediction. Ghent: University Press Zelzate (2015).

[16] Bös K. Deutscher Motorik-Test 6-18: (DMT 6-18) Manual und Internetbasierte Auswertungssoftware. Hamburg: Feldhaus Edition Czwalina (2016). p. 96.

[17] Siener M Hohmann A. Talent orientation: the impact of motor abilities on future success in table tennis. Ger J Exerc Sport Res. (2019) 49:232–43. 10.1007/s12662-019-00594-1

[18] Utesch T Strauß B Tietjens M Büsch D Ghanbari M-C Seidel I. Die Überprüfung der Konstruktvalidität des Deutschen Motorik-tests 6–18 für 9- bis 10-jährige. Zeitschrift für Sportpsychologie. (2015) 22:77–90. 10.1026/1612-5010/a000143

[19] Hohmann A Fehr U Voigt L. Heute im talentpool—in Hamburg auf dem podium. Leistungssport. (2015) 45:5–11.

[20] Hohmann A Siener M He R. Prognostic validity of talent orientation in soccer. Ger J Exerc Sport Res. (2018) 48:478–88. 10.1007/s12662-018-0549-5

[21] Teunissen JW Faber IR de Bock J Slembrouck M Verstockt S Lenoir M A machine learning approach for the classification of sports based on a coaches’ perspective of environmental, individual and task requirements: a sports profile analysis. J Sports Sci. (2023):1–10. 10.1080/02640414.2023.2271706 38105561

[22] Christensen MK. “An eye for talent”: talent identification and the “practical sense” of top-level soccer coaches. Sociol Sport J. (2009) 26:365–82. 10.1123/ssj.26.3.365

[23] Johnson MB Castillo Y Sacks DN Cavazos J Edmonds WA Tenenbaum G. “Hard work beats talent until talent decides to work hard”: coaches’ perspectives regarding differentiating elite and non-elite swimmers. Int J Sports Sci Coach. (2008) 3:417–30. 10.1260/174795408786238579

[24] Johnston K Baker J. Waste reduction strategies: factors affecting talent wastage and the efficacy of talent selection in sport. Front Psychol. (2020) 10:2925. 10.3389/fpsyg.2019.02925 31998188 PMC6967295

[25] Schorer J Rienhoff R Fischer L Baker J. Long-Term prognostic validity of talent selections: comparing national and regional coaches, laypersons and novices. Front Psychol. (2017) 8:1146. 10.3389/fpsyg.2017.01146 28744238 PMC5504223

[26] Bergkamp TL Niessen AS den Hartigh RJ Frencken WG Meijer RR. Methodological issues in soccer talent identification research. Sports Med. (2019) 49:1317–35. 10.1007/s40279-019-01113-w 31161402 PMC6684562

[27] den Hartigh RJ Niessen AS Frencken WG Meijer RR. Selection procedures in sports: improving predictions of athletes’ future performance. Eur J Sport Sci. (2018) 18:1191–8. 10.1080/17461391.2018.1480662 29856681

[28] Teunissen JW Ter Welle S Platvoet S Faber I Pion J Lenoir M. Similarities and differences between sports subserving systematic talent transfer and development: the case of paddle sports. J Sci Med Sport. (2021) 24:200–5. 10.1016/j.jsams.2020.09.005 32972845

[29] Roberts AH Greenwood DA Stanley M Humberstone C Iredale F Raynor A. Coach knowledge in talent identification: a systematic review and meta-synthesis. J Sci Med Sport. (2019) 22:1163–72. 10.1016/j.jsams.2019.05.008 31133481

[30] Roberts AH Greenwood D Humberstone C Raynor AJ. Pilot study on the reliability of the coach’s eye: identifying talent throughout a 4-day cadet judo camp. Front Sports Act Living. (2020) 2:596369. 10.3389/fspor.2020.596369 33345177 PMC7739670

[31] Robertson K Pion J Mostaert M Norjali Wazir MR Kramer T Faber IR A coaches’ perspective on the contribution of anthropometry, physical performance, and motor coordination in racquet sports. J Sports Sci. (2018) 36:2706–15. 10.1080/02640414.2018.1441941 29465332

[32] Bös K. Handbuch Motorische Tests: Sportmotorische Tests, Motorische Funktionstests, Fragebögen zur Körperlich-sportlichen Aktivität und Sportpsychologische Diagnoseverfahren. Göttingen: Hogrefe (2017). p. 899.

[33] Sieghartsleitner R Zuber C Zibung M Conzelmann A. “The early specialised bird catches the worm!” - a specialised sampling model in the development of football talents. Front Psychol. (2018) 9:188. 10.3389/fpsyg.2018.00188 29515500 PMC5826374

[34] Streiner DL. Starting at the beginning: an introduction to coefficient alpha and internal consistency. J Pers Assess. (2003) 80:99–103. 10.1207/S15327752JPA8001_18 12584072

[35] Cicchetti DV. Guidelines, criteria, and rules of thumb for evaluating normed and standardized assessment instruments in psychology. Psychol Assess. (1994) 6:284–90. 10.1037/1040-3590.6.4.284

[36] Benjamini Y Hochberg Y. Controlling the false discovery rate—a practical and powerful approach to multiple testing. J Royal Stat Soc B (Methodological). (1995) 57:289–300. 10.1111/j.2517-6161.1995.tb02031.x

[37] Pion J Segers V Fransen J Debuyck G Deprez D Haerens L Generic anthropometric and performance characteristics among elite adolescent boys in nine different sports. Eur J Sport Sci. (2015) 15:357–66. 10.1080/17461391.2014.944875 25143133

[38] Seifert L Papet V Strafford BW Coughlan EK Davids K. Skill transfer, expertise and talent development: an ecological dynamics perspective. Mov Sport Sci/Sci Mot. (2018) 19:39–49. 10.1051/sm/2019010

[39] Charbonnet B Conzelmann A. Talent development in childhood: early specialization or sampling? From an either… or… question to a 2x2x3 question cuboid. Int J Sports Sci Coach. (2023) 27:1–17. 10.1177/17479541231197225

[40] Till K Baker J. Challenges and possible solutions to optimizing talent identification and development in sport. Front Psychol. (2020) 11:664. 10.3389/fpsyg.2020.00664 32351427 PMC7174680

